# A survey of motif finding Web tools for detecting binding site motifs in ChIP-Seq data

**DOI:** 10.1186/1745-6150-9-4

**Published:** 2014-02-20

**Authors:** Ngoc Tam L Tran, Chun-Hsi Huang

**Affiliations:** 1 Department of Computer Science and Engineering, University of Connecticut, 371 Fairfield Way, Unit 4155, Storrs, CT 06269, USA

**Keywords:** Motif finding Web tool, Peak calling, Binding site, Over-represented motif, ChIP-Seq

## Abstract

**Abstract:**

ChIP-Seq (chromatin immunoprecipitation sequencing) has provided the advantage for finding motifs as ChIP-Seq experiments narrow down the motif finding to binding site locations. Recent motif finding tools facilitate the motif detection by providing user-friendly Web interface. In this work, we reviewed nine motif finding Web tools that are capable for detecting binding site motifs in ChIP-Seq data. We showed each motif finding Web tool has its own advantages for detecting motifs that other tools may not discover. We recommended the users to use multiple motif finding Web tools that implement different algorithms for obtaining significant motifs, overlapping resemble motifs, and non-overlapping motifs. Finally, we provided our suggestions for future development of motif finding Web tool that better assists researchers for finding motifs in ChIP-Seq data.

**Reviewers:**

This article was reviewed by Prof. Sandor Pongor, Dr. Yuriy Gusev, and Dr. Shyam Prabhakar (nominated by Prof. Limsoon Wong).

## Open peer review

Reviewed by Prof. Sandor Pongor, Dr. Yuriy Gusev, and Dr. Shyam Prabhakar (nominated by Prof. Limsoon Wong). For the full reviews, please go to the Reviewers’ comments section.

## Introduction

The Next Generation Sequencing (NGS) technology has revolutionized the genetics studies of RNA-Seq for transcriptome analysis and ChIP-Seq for DNA-proteins interaction [1]. ChIP-Seq has become the method of choice for genome-wide characterization of transcription factor binding, polymerase binding, and histone modifications [2]. The identification of binding sites by transcription factors, polymerase, or histone modification marks plays a crucial role for identifying the regulatory elements that regulate the gene expression. Several peak calling tools have been developed for detecting the binding sites in ChIP-Seq. These tools identify the binding sites using a common method of calculating the density of read counts called peaks. Peak calling tools output the list of peak sequences in various sizes and different formats. The actual binding sites are often short sequences embedded in these peak sequences. The actual DNA region, which interacts with a single transcription factor (TF), typically ranges from 8–10 to 16–20 bp [2]. In addition, the binding sites for TF in ChIP-Seq are usually located in close proximity of the summit points of the peaks [3]. Zambelli et al. describes the TFs bind to the DNA in a sequence specific way that they recognize sequences that are similar but not identical and differ by only some nucleotides from one another [2]. Thus, identifying the conserved motifs in these sequences reveals the same TF binding to them. Motif finding is one of the well-known studies in Bioinformatics. Many tools have been developed for motif finding. Recent motif finding development provides user-friendliness via Web interface. In this work, we surveyed nine motif finding Web tools that are capable for finding motifs in ChIP-Seq data. These tools are listed in Table 1.

**Table 1:** A summary of motif finding web tools

**Web Tool**	**Pipeline**	**Accept File Format**	**Maximum File Size**	**Maximum Sequence Length**	**P-Value Option**	**Motif’s Size Option**	**# of Motifs Option**	**Ref. Database**	**Web Tool**	**Approach**	**Ref. Database Option**	**Ref. Genome Option**	**Log in Required**	**Email Required**	**User Account Option**	**Published Year**	**Current Version**	**Ref. #**
MEME	No	Fasta	= 60000 characters	< 1000 bp	No	Yes	Yes	JASPAR, BLOCKS, UniProbe, …., user database	MEME	Implemented Multiple EM	No	No	No	Yes	No	2006	4.9.1	[4]
GLAM2	No	Fasta	= 60000 characters	= 10000 bp	No	No	No	JASPAR, UniProbe, …., user database	GLAM2	Implemented novel Gapped Local Alignment of Motifs algorithm	No	No	No	Yes	No	2008	4.9.1	[5]
CisFinder	No	Fasta, plain text delimited	Unspecified	= 50 Mb	FDR option	No	Yes	JASPAR, CisView, …., user database	CisFinder	Implemented novel CisFinder algorithm	Yes	No	Optional	Optional	Optional	2009	Unspecified	[6]
W-ChIPMotifs	Yes	Fasta	Unspecified	Unspecified	No	No	No	JASPAR, TRANSFAC, …., user database	W-ChIPMotifs	Used existing ChIPMotifs program and incorporated other existing tools: MEME, MaMF, and Weeder	No	Human and Mouse only	No	Yes	No	2009	Unspecified	[7]
CompleteMOTIFs	Yes	Bed, fasta, gff	= 500000 bp for MEME, Weeder, = 5000000 for ChIPMunk	Unspecified	Yes	Yes for MEME	No	JASPAR, TRANSFAC	CompleteMOTIFs	Integrated existing tools: MEME, Weeder, and ChIPMunk	Yes	Yes	Optional	Optional	Optional	2011	Unspecified	[8]
DREME	No	Fasta	Unspecified	Unspecified	E-value option	No	No	JASPAR, UniProbe, …., user database	DREME	Implemented novel Discriminative Regular Expression Motif Elicitation algorithm (DREME)	No	No	No	Yes	No	2011	4.9.1	[9]
MEME-ChIP	Yes	Fasta	Unlimited	Unlimited	E-value option	Yes	Yes	JASPAR, UniProbe, …., user database	MEME-ChIP	Integrated existing tools: MEME and DREME	No	No	No	Yes	No	2011	4.9.1	[10]
RSAT peak-motifs	Yes	Raw, multi, tab, fasta, wconsensus, IG	Unlimited	Unlimited	No	Yes	Yes	JASPAR, UniProbe, DMMPMM, RegulonDB, …, user database	RSAT peak-motifs	Implemented RSAT oligo-analysis, RSAT dyad-analysis, RSAT local-word analysis, MEME, ChlPMunk	Yes	No	No	Optional	No	2012	Unspecified	[11]
PScanChIP	No	Bed	Unlimited	100 -150 bp	No	No	No	JASPAR, TRANSFAC	PScanChIP	Used existing Pscan algorithm	Yes	Yes	No	No	No	2013	1.0	[3]

## Review

### General approaches for motif finding

Motifs are short sequences of a similar pattern found in sequences of DNA or protein. Consider *t* input nucleotide sequences of length *n* and an array *s* (*s*_1_, *s*_2_, *s*_3_, …, *s*_*t*_) of starting positions with each position comes from each sequence. An alignment matrix is a matrix of *t* × *l*, which contains *t* sequences of starting positions from each sequence with length *l* where *l* is the size of an *l*-mer. A profile matrix is a matrix of 4 × *l* containing 4 rows for four nucleotides (A, C, G, T) and *l* columns. Each entry in the profile matrix is the frequency of each nucleotide in the alignment matrix. The consensus score is the sum of highest frequencies from each column in the profile matrix. The motif finding problem can be stated simply as follows. Given *t* input nucleotide sequences of length *n*, we want to find a set of *l*-mers with one from each sequence such that they maximize the consensus score. Thus, we need to consider all (*n* - *l* + 1)^*t*^ possible starting positions or candidates for motifs. That is the number of candidates for motifs is exponential of the number of input sequences. In fact, motif finding is an NP-complete problem [12]. There are several different approaches for finding motifs such as profiles, consensuses, projection, graph representations, clustering, and tree-based [2,13,14].

#### ***Profiles***

This approach uses a Position Weight Matrix (PWM) for representing the frequency of four possible nucleotides appearing in each position of the motif [13]. The PWM is a matrix of 4 × *l* containing 4 rows for four nucleotides (A, C, G, T) and *l* columns where *l* is the size of the motif. Using a PWM, the most likely location of the motif within each sequence can be calculated [13].

Some examples of profiles-based algorithms can be found in [15-19].

#### ***Consensuses***

In this approach, a consensus string is formed for each profile, which is constructed for each of the possible sets of starting locations in the alignment of the sequences. The best consensus with highest score is chosen to describe the motifs in the sequences [13].

Some examples of consensus-based algorithms are WINNOWER [20], CONSENSUS [21], and ProfileBranching [22].

#### ***Projection***

This approach solves the (*l*, *k*) motif problem where each instance of a motif of length *l* differs from the original motif by exactly *k* positions. These *k* positions are used as hashing functions for all possible contiguous sequences of *l* nucleotides. The potential motif sequences are put in the buckets based on their hashing functions. If the number of *l*-mers hash to the same bucket exceeds a threshold, they are considered as good candidates for motifs. The algorithm searches these buckets for the candidates of motifs [13].

Some examples of projection-based algorithms are PROJECTION [23] and Uniform Projection Motif Finder (UPMF) [24].

#### ***Graph representations***

This approach recasts the motif finding problem into graph solving problem in which nodes correspond to substrings of input sequences and edges connecting nodes correspond to similar substrings [2]. Thus, the motifs can be found by detecting cliques [25] or maximum density sub-graphs [26].

#### ***Clustering***

The motif finding can be transformed into finding the clusters in which the substrings of the input sequences forming the motif should be clustered together, and the rest should belong to a background cluster [2]. Thus, the cluster finding can be solved using appropriate clustering strategies like self organizing maps [27,28].

#### ***Tree-based***

This approach models the motif finding using tree-based data structure and uses tree-based algorithms to solve the motif detection. Al-Turaiki et al. modelled the motif finding problem using Trie data structure and transformed the motif finding into mining frequent patterns in large datasets [14]. Mohapatra et al. transformed the motif finding into generalized suffix tree and developed a tree-based algorithm for finding motifs [29].

### Motif finding Web tools

#### ***General features of motif finding Web tools***

The implementation of motif finding Web tools generally falls into two categories. The first category is pipeline implementation, which incorporates existing tools into a Web tool/Web service. The second category involves implementing novel algorithms into a Web tool/Web service. Generally, motif finding Web tools allow uploading input sequences of DNA, protein, or binding sites. The users can customize the motif finding strategy before submitting the request. The results can be displayed on the browser or can be downloaded. However, different motif finding Web tools provide different customizations for finding motifs as well as provide different result formats. Some Web tools have restrictions on the size of input sequence, the number of peaks, or the size of upload file. Others provide flexibility for input file formats and allow creating an account for storing the results on the server. Some Web tools require email address for notifying the result. All motif finding Web tools have their own features for verifying discovered motifs with one or more motif reference databases such as JASPAR [30], TRANSFAC [31], CisView [32], UniProbe [33], and user’s reference. Some Web tools allow selecting one or more motif reference databases while others use their own pre-selected references. Some Web tools provide options for selecting the reference genome, motif size, and the number of motifs to return.

In the following section, we observe the features, approach, strengths and weaknesses of each motif finding Web tool.

#### ***MEME***

MEME [4] (Multiple EM for Motif Elicitation) is a Web service available on MEME suite [34]. MEME allows running motif detection on its Website or through several mirror sites. It can be downloaded and installed locally. MEME is a de novo motif finding tool, which was designed for finding un-gapped motifs in unaligned DNA or protein sequences. MEME only accepts ≤ 60,000 characters in the input file, which must be in fasta format. The input sequence’s length should be ≤ 1,000 bp and as short as possible. MEME suggests removing duplicate sequences and sequences with low information that may not contain the motif prior to running the motif finding. MEME allows specifying the length of the motif and the number of motifs to return. It also allows entering the number of sites for each motif if there is a prior knowledge about the number of occurrences that the motif has in the dataset. MEME requires specifying how the user believes the occurrences of the motifs are distributed among the sequences, for example, zero or one per sequence. MEME includes the option in the results on the browser for verifying discovered motifs with the reference database. Its initial version allowed verifying discovered motifs with JASPAR [30] or BLOCKS [35] reference database. In its later versions, MEME allows using TOMTOM [36] for verifying discovered motifs. MEME requires email address for notifying the results. It does not allow either creating an account or storing the results on the server. MEME includes other options such as performing discriminative motif discovery, uploading file containing a background Markov model, searching a given strand or both given strand and reverse strand, and looking for palindromes [4].

A summary of MEME’s features can be found in Table 1.

MEME provides three different output formats: HTML, XML, and text. The output shows the motifs as local multiple alignments of the input sequences. It allows sending motifs to MAST [37] Web server for searching the sequences that match discovered motifs. MEME also provides other options in HTML output for forwarding one or all motifs to other Web-based programs for further analysis. For each motif, MEME outputs E-value, number of sites found, motif’s logo, motif’s blocks format, motif’s block diagrams, position-specific scoring matrix, position-specific probability matrix, and so on [4].

MEME algorithm extends the expectation maximization (EM) algorithm [38]. The EM algorithm for motif finding presented by Lawrence et al. has the following drawbacks. It is not clear how to select the starting point and when to stop trying different starting points. It assumes there is exactly one appearance of the shared motif appearing in each sequence of the dataset but this is not always the case. MEME algorithm overcomes these limitations. MEME selects starting points based on all sub-sequences of sequences in the training dataset. It also eliminates the assumption of the shared motif appearing in each sequence. MEME removes the appearances of a motif after it is discovered and keeps searching for additional shared motifs in the dataset [38].

Because MEME erases previous discovered motifs when it searches for new motifs, MEME can only model a single motif at a time and it does not detect alternative binding motifs, which are motifs for co-factors.

#### ***GLAM2***

We included this tool for finding consensus motifs [39] as there is a possibility of having deletion or insertion (indels) in the binding sites of the peak sequences from ChIP-Seq.

GLAM2 [5] (Gapped Local Alignment of Motifs) is a de novo motif finding Web tool, which was designed for finding motifs with indels in unaligned DNA or protein sequences. The tool can be installed locally or can be run on MEME suite [34]. GLAM2 only accepts input sequences in fasta format with ≤ 60,000 characters in the input file. GLAM2 contains several features that can be customized for the motif finding. These features include aligned columns, alignment replicates, iterations without improvement, insertion, deletion, shuffling, and examining forward and reverse strands. GLAM2 requires email address for notifying the results. However, it does not allow either creating an account or storing the results on the server [5].

A summary of GLAM2’s features can be found in Table 1.

GLAM2 provides three different output formats: HTML, text, and MEME text format. It outputs the best motifs found with their start and end positions, sites, strand, marginal score, and motif’s logo. GLAM2 has a scanning method called GLAM2SCAN, which is used for scanning the alignment of the motif results against sequence databases. This method is also included in the HTML output. GLAM2’s HTML output contains an option for verifying discovered motifs with the references using TOMTOM [36] program. Other options in the HTML output include viewing alignment, viewing Position Specific Probability Matrix (PSPM), and finding replications that are similar to the best motif found [5].

The PSPM is a 4 × *l* matrix containing 4 rows for four nucleotides (A, C, G, T) and *l* columns where *l* is the size of the motif. Each entry in the matrix is the frequency of a nucleotide in the multiple alignments of the sequences. This frequency is represented by a probability value.

GLAM2 implemented a generalization of the gapless Gibbs sampling algorithm. It examines the input sequences and returns an alignment of segments of these sequences. Each sequence appears in at most one segment of the alignment. GLAM2 assumes a motif is defined by residue preferences at certain positions called key positions. However, the key positions can be deleted or the residues can be inserted between these key positions in a particular motif. GLAM2 implemented a scoring scheme for alignments in which any identical residues or similar residues alignment happens in the same key position is rewarded while deletions and insertions are penalized. However, the penalty is not severe if deletions and insertions constantly occur in the same locations. Using this scoring scheme, GLAM2 calculates the marginal score, which reflects how well each segment matches the other segments. GLAM2 finds a motif alignment with maximum score using the scoring scheme. Because the number of possible alignments is too large, GLAM2 uses a heuristic optimization method called simulated annealing for finding the motif alignment with maximum score. This method takes an initial alignment and constantly makes changes to it. These changes increase the score and also decrease the score. GLAM2 performs two types of changes called site sampling and column sampling. The changes are applied until the score fails to improve. To verify high score motif found, the whole procedure is repeated for a number of times from different random starting alignments. GLAM2’s performance can also be controlled by several given parameters as described above [5].

GLAM2 is time consuming and its running time scales linearly with the sequence’s length. GLAM2 works best for small datasets and short motifs. It is difficult for GLAM2 to analyze sequences longer than a few thousand residues and it is impractical for GLAM2 to analyze sequences that are ≥ 10,000 bp [5].

GLAM2 can only model a single motif at a time and it does not detect alternative binding motifs.

#### ***CisFinder***

CisFinder [6] is a de novo motif finding Web tool for finding over-represented short DNA motifs. It implemented the novel CisFinder algorithm. The tool accepts input sequences in fasta format and plain text delimited format. CisFinder accepts four main file types: sequences, motifs, search results, and repeats. It was designed for processing large input dataset up to 50 Mb. CisFinder allows uploading the control file or using the public control file provided by the tool. CisFinder provides several analysis tools such as identifying motifs, improving motifs, clustering motifs, comparing motifs, showing motif, searching motif, and showing search results. It allows downloading and deleting each of four main file types. CisFinder provides several different parameters for customizing the motif finding. It does not allow specifying motif size but it allows selecting motif reference databases such as JASPAR [30], CisView [32], or user’s reference. CisFinder allows using Guest account or setting up a user account for using the tool. Registered users can store the results on the server while Guest user has only one full session [6].

A summary of CisFinder’s features can be found in Table 1.

CisFinder’s output can be in HTML and text formats. The output contains elementary motifs and cluster motifs with both can be saved or downloaded. The elementary motifs are listed by name, logo, pattern, frequency, enrichment ratio, information content of motif, score, FDR, and so on. Motifs cluster is listed by name, logo, pattern, number of motifs in cluster, frequency, enrichment ratio, information content of motif, score, FDR, palindrome, method of motif clustering, and so on [6].

CisFinder algorithm is based on the estimation of position frequency matrices (PFMs). This estimation is calculated from *n*-mer word counts in the test set and control set of sequences. CisFinder contains five main features. First, the algorithm is based on detecting over-represented short words in a sequence and clustering them. Second, the algorithm examines words with gaps and expands PFMs over the gaps and neighboring regions. Third, it uses real control sequences to compare against test sequences for processing repeat regions without removing repeat sequences because TF binding sites are often located in repeat regions. Fourth, it implements exhaustive searches for all over-represented DNA motifs in a single run and combines motifs only at the clustering step. Finally, it includes several other functions such as comparing motifs with reference databases, searching for motifs that match PFMs, visualizing sequences and TF binding motifs with CisView [32] or UCSC genome browser [40], and extracting of sequence fractions and subsets of sequences [6].

CisFinder provides flexibility for input file formats and file types. It can process large datasets and provides several tools for motif analysis. CisFinder algorithm can accurately identify PFMs of TF binding motifs [6]. CisFinder runs much faster than MEME [4], Weeder [41], and RSAT [6,42]. It can detect alternative binding motifs as well as binding motifs of potential co-factors. Finally, it can find motifs with a low level of enrichment [6].

#### ***W-ChIPMotifs***

W-ChIPMotifs [7] is a de novo motif finding Web tool for ChIP-based high throughput data. It only accepts input sequences in fasta format. W-ChIPMotifs does not specify either the maximum input file size or the maximum sequence length. The tool does not have options for specifying motif size and number of motifs to return. W-ChIPMotifs incorporated STAMP [43] tool for inferring phylogenetic information and verifying discovered motifs with the reference databases. It requires specifying human or mouse species, user’s name, email, and transcription factor before submitting the request. The tool allows supplying the control file. However, it does not allow either creating an account or storing the results on the server [7].

A summary of W-ChIPMotifs’s features can be found in Table 1.

The outputs of W-ChIPMotifs contain two files in PDF format via email only. One file contains found motifs and the other contains matched similar motifs from STAMP. The discovered motifs are listed by name, logo, confidence level, PWMs, core and PWM scores, P-values, and Bonferroni correction P-value. The matching motifs from STAMP are listed by name, E-value, alignment, and logo. A phylogenetic tree for matching motifs is also included [7].

W-ChIPMotifs is based on the previous ChIPMotifs program [7]. The tool is a pipeline system, which incorporated three motif finding tools: MEME [4], MaMF [44], and Weeder [45] for motif detection [7]. W-ChIPMotifs optimizes the significance of found motifs using bootstrap re-sampling method and Fisher test. It identifies about less than 10 candidate motifs for constructing *n* PWMs for each candidate motif. Then, it uses a bootstrap re-sampling method to infer the optimized PWM scores. If the control data is not supplied W-ChIPMotifs uses the default control dataset based on the species selected by the user. It generates negative control dataset by randomizing the input sequences with each sequence for 100 times. The generated negative control dataset no longer corresponds to the original sequences but it shares the same nucleotide frequencies and it is used for scanning the identified motifs. W-ChIPMotifs uses Fisher test and P-value for identifying the significant cutoff for the scores [7].

W-ChIPMotifs can only model a single motif at a time and it does not detect alternative binding motifs. However, it combines three existing motif finding tools for maximizing the chance obtaining true motifs.

#### ***DREME***

DREME [9] (Discriminative Regular Expression Motif Elicitation) is a motif finding Web tool available from MEME suite [34]. It was designed for finding short (≤ 8 bases), core DNA-binding motifs of eukaryotic TFs and it is able to process very large ChIP-Seq datasets [9]. DREME is capable for finding binding motifs for cofactor TFs. It only accepts input sequences in fasta format. DREME allows setting E-value cutoff but it does not allow specifying motif size. DREME includes the option in the output for verifying found motifs with reference databases using TOMTOM [36] program. DREME requires email address for notifying the results. It requires selecting comparison source, which is set to shuffled sequences by default. It allows specifying the type of strand to use. DREME does not allow either creating an account or storing the results on the server [9].

A summary of DREME’s features can be found in Table 1.

DREME provides three different output formats: HTML, XML, and text. The found motifs are listed by name, logo, and E-value. The motif’s details include number of positive and negative strands matching that motif, P-value, E-value, and enriched matching words for that motif. DREME allows submitting discovered motifs to other programs within MEME suite [34] for further analysis. The found motif can be downloaded as a position weight matrix or a custom logo [9].

DREME algorithm is based on a simplified form of regular expression. Its motif detection is exhaustive for exact words and heuristic for words with wildcards. To identify the significant, discriminative motifs, the algorithm uses Fisher’s Exact test for calculating the significance of relative enrichment of each motif in two sets of sequences. One set is the set of ChIP-Seq peak regions and the other is either similar data from a different ChIP-Seq experiment or shuffled versions of the first sequences. The algorithm counts only the number of sequences containing a motif in each dataset. When the motif with highest significance is found, all of its non-overlapping occurrences in the first set of sequences are aligned to create a position specific probability matrix. To find multiple, non-redundant motifs in a set of sequences, the algorithm erases the best motif found by setting all its occurrences to a special letter that cannot match any motif. Then, the algorithm repeats the search for motifs [9].

DREME is much faster than MEME [4], Weeder [41], and NestedMICA [9,46]. Its runtime scales linearly with the size of the dataset [9].

#### ***MEME-ChIP***

MEME-ChIP [10] is a Web service designed for analyzing ChIP-Seq datasets and it is available from MEME suite [34]. MEME-ChIP provides several analysis tools such as motif discovery, motif enrichment analysis, motif visualization, binding affinity analysis, and motif identification. MEME-ChIP is a pipeline system, which incorporated MEME and DREME into a Web service. MEME-ChIP only accepts input sequences in fasta format. It does not have restrictions on the size of input sequence and the number of upload sequences. Thus, MEME-ChIP can analyze very large ChIP-Seq datasets. It allows setting E-value cutoff as well as selecting motif size and number of motifs to return. MEME-ChIP allows verifying found motifs with several motif reference databases. It provides universal options, MEME options, DREME options, and CentriMo [47] options for customizing the motif detection. MEME-ChIP requires email address for notifying the results. However, it does not allow either creating an account or storing the results on the server [10].

A summary of MEME-ChIP’s features can be found in Table 1.

MEME-ChIP provides three different output formats: HTML, XML, and text. The output can be viewed in MEME output format, DREME output format, as well as in CentriMo [47] and TOMTOM [36] report formats [10].

MEME-ChIP incorporated two complementary motif finding algorithms MEME and DREME [10]. MEME implemented multiple EMs while DREME used the regular expression approach. DREME is capable for detecting very short motifs that are not found by MEME. MEME-ChIP used TOMTOM [36] for verifying discovered motifs by MEME and DREME with the reference databases [10]. MEME-ChIP also used AME algorithm [48] for detecting very low levels of enrichment of binding sites for motif enrichment analysis [10]. MEME-ChIP used MAST [37] and AMA [49] algorithms for visualizing motifs as well as for binding strength analysis [10,48].

#### ***CompleteMOTIFs***

CompleteMOTIFs [8] is a de novo motif finding Web tool, which was designed for finding over-represented transcription factor binding motifs from ChIP-Seq. CompleteMOTIFs is a pipeline system, which incorporated MEME [4], Weeder [45], and ChIPMunk [50] into a Web tool. It accepts input sequences in fasta, BED, and GFF formats. CompleteMOTIFs accepts file’s size ≤ 500,000 bp for MEME and Weeder. It accepts ≤ 5,000,000 bp in input file for ChIPMunk. CompleteMOTIFs allows selecting motif reference database as well as allows supplying user’s reference in Position Specific Scoring Matrices. It also requires specifying the type of the background sequence and the reference genome used in the motif finding. Other options for customizing the motif detection include setting P-value cutoff, specifying the types of nucleotides shuffling, and the number of times for nucleotides shuffling. CompleteMOTIFs allows specifying motif size for running MEME only. It does not allow specifying the number of motifs to return. CompleteMOTIFs allows using Guest account or setting up a user account for using the tool. Registered users can store the results on the server. CompleteMOTIFs also provides annotation analysis and eight boolean logic operations for file manipulation. It also provides two utilities: convert BED to fasta, and convert fasta to BED [8].

A summary of CompleteMOTIF’s features can be found in Table 1.

CompleteMOTIF’s output can be in both HTML and text formats. The output can be viewed in MEME [4], Weeder [45], and ChIPMunk [50] formats depending on the selections when submitting the request. The motif results can be verified with JASPAR [30] and TRANSFAC [31] databases using Patser [21] scanning method. The top 10 motifs with their logos can be viewed on the browser. The tool also shows the motif clustering result from STAMP [43]. All results can be downloaded in a zip file [8].

CompleteMOTIF incorporated three existing motif finding tools into a Web tool. However, the results are specific to each tool selected by the user. Each tool has its own approach for finding motifs. MEME used the multiple EMs algorithm [4]. Weeder implemented a suffix tree based exhaustive enumeration algorithm [45]. ChIPMunk implemented an iterative algorithm that combines greedy optimization with bootstrapping [50].

#### ***RSAT peak-motifs***

Peak-motifs [11] is a pipeline system for finding motifs in ChIP-Seq data. It can be used as a stand-alone application and Web services. peak-motifs provides several selective categories for customizing the motif detection as follows [11].

##### 

**Uploading input** Peak-motifs accepts different types of input sequences such as raw, multi, tab, fasta, wconsensus, and IG formats. The input sequences can be uploaded in a .gz compressed file. peak-motifs can also take the input from other Web server via URL. The input sequences can be masked into lowercase, uppercase, or non-dna. peak-motifs does not have limitations for the size of the sequence and the number of peaks in the input. It also allows uploading the control sequences [11].

##### 

**Reducing peak sequences** Peak-motifs provides flexibility for selecting the number of top sequences to retain for the motif finding. It allows reducing peak sequences by a number of base pairs on each side of the peak center for the motif detection [11].

##### 

**Motif discovery parameters** Peak-motifs provides options for finding over-represented words, words with a positional bias, words with local over-representation, and over-represented spaced word pairs. It allows selecting oligomer lengths 6, 7, and 8 characters. peak-motifs also includes several selections for Markov order of the background model. The users can select between 1 to 10 motifs to return per algorithm as well as selecting a single strand or both strands for the motif detection. peak-motifs provides several options for selecting different reference databases including user’s database and known reference motifs for verifying discovered motifs [11].

##### 

**Locating and visualizing motifs** Peak-motifs allows searching putative binding sites in the peak sequences. It includes several options for selecting Markov order of the background model for sequence scanning. It also allows visualizing peaks and sites on the genome browser [11].

##### 

**Output option** Peak-motifs provides two output options: displaying the results on the browser or emailing the results to the user. The latter requires user’s email address [11].

A summary of peak-motifs’s features can be found in Table 1.

All motif results can be downloaded in a zip file. All matrices can be downloaded in TRANSFAC format. peak-motifs displays detailed results in several different categories such as sequence compositions and statistics, number of discovered motifs by algorithm, number of discovered motifs with motif comparison, individual motifs and their matrices, motif locations or sites, and motif comparisons [11].

Peak-motifs is a computational pipeline that incorporated several algorithms. The algorithms used for motif finding are RSAT dyad-analysis [51], RSAT local-word analysis [52], MEME [4], and ChIPMunk [11,50]. peak-motifs also implemented the pattern matching algorithm called matrix-scan-quick from RSAT [11,53]. It used RSAT compare-motifs algorithm for motif comparison. The implementation of motif finding relies on a combination of tried and tested algorithms, which integrated in the software suite RSAT. The motif finding also used complementary criteria for detecting the motifs [11].

#### ***PscanChIP***

PscanChIP [3] is a motif finding Web tool for ChIP-Seq data. It only accepts input sequences in BED format. PscanChIP assumes that the region is centered on the point of maximum enrichment within the peak and it only analyzes 150 bp around the summit for that region. It does not provide options for selecting motif size and the number of motifs to return. PscanChIP requires selecting human or mouse species with its associated assembly. It allows selecting the background model and the motif reference databases such as JASPAR [30], TRANSFAC [31], and user’s database. PscanChIP does not allow either creating an account or storing the results on the server [3].

A summary of PscanChIP’s features can be found in Table 1.

PscanChIP’s output can be in HTML and text formats. The results include several categories such as binding profile name, binding profile ID, local enrichment P-value, local over- or under- representation, global enrichment P-value, global over- or under- representation, Spearman correlation coefficient, preferred position, position bias P-value, and so on. For each matrix in the results, PscanChIP shows matrix’s detailed information, its position weight matrix (PWM), motif’s logo, and all occurrences [3].

PscanChIP is based on the previous Pscan tool for promoter analysis. It computes the global enrichment, which is used for identifying motifs that are overrepresented in the regions. It also calculates local enrichment, which is used for identifying motifs with significant preference for binding within the regions. In addition, PscanChIP evaluates motif positional bias within the input regions. It can identify the actual binding sites for the TF and the secondary motifs corresponding to other TFs that tend to bind the same region [3].

### Peak calling tools

There are many factors, which can affect the result of the motif finding such as quality of the antibody used, read length, sequencing error, read mapping procedure, peak caller, and so on. Here we only mentioned the closest influence factor, which is the peak calling tool in this section. We recommend the users to select the peak calling tool that is relevant to the type of research being conducted. We also provided a summary for a number of peak calling tools in Table 2. Besides, the control data is important for background model validation. Thus, it is better to run the peak calling tool using both input and control data.

**Table 2:** A summary of peak calling tools

**Tool**	**Algorithm**	**Approach**	**Published year**	**Language**	**Operating system**	**Software features**	**Latest release version**	**Latest release year**	**Website**	**Maintenance**	**Ref. #**	
BayesPeak	BayesPeak algorithm	Used Hidden Markov model (HMM) for finding peaks	2011	R and C	Linux, Windows, and Mac OS X	Support multicore	1.12.0	N/A	http://compbio.sysbiol.cam.ac.uk/Resources/BayesPeak/csbayespeak.html	Yes	[54]	
BroadPeak	Maximal-segment algorithm, Gibbs sampling algorithm, Ruzzo–Tompa algorithm	Probabilistic model	2013	R	N/A	N/A	One version	2013	http://jordan.biology.gatech.edu/page/software/broadpeak/	Yes	[55]	
CisGenome	Two-pass algorithm	Implemented a modular design, use sliding window for peak detection	2008	C, C++	Windows, Mac, and Linux	Stand-alone system, command mode and GUI	v2.0	2011	http://www.biostat.jhsph.edu/~hji/cisgenome/	Yes	[56]	
DROMPA (DRaw and observe Multiple enrichment profiles and annotation)	Sliding window	Two-step procedure, DROMPA peak-calling program	2013	ANSI-C	Linux	N/A	1.4.0	2013	http://www.iam.u-tokyo.ac.jp/chromosomeinformatics/rnakato/drompa/	Yes	[57]	
F-Seq	F-Seq density estimation algorithm	Kernel density estimation	2008	Java	Unix, Linux	N/A	1.84	2011	http://fureylab.web.unc.edu/software/fseq/	Yes	[58]	
FindPeaks	Used directional reads module for identifying peaks	Implemented a modular architecture	2008	Java	Linux, Windows, and Mac OS X	Command line	4.0	N/A	http://vancouvershortr.sourceforge.net/	Yes	[59]	
GEM (Genome wide Event finding and Motif discovery)	Genome wide event finding and motif discovery (GEM)	Probabilistic model	2012	Java	N/A	Stand-alone software	2.3	2013	http://cgs.csail.mit.edu/gem/	Yes	[60]	
GLITR (GLobal Identifier of Target Regions)	GLITR algorithm	Used ChIP-Seq Peak Finder framework	2009	Perl and Python	N/A	N/A	N/A	N/A	N/A	N/A	[61]	
GLMNB (Negative binomial generalized linear model)	Sliding window	Generalized Linear Model with Negative binomial distribution	2012	N/A	N/A	N/A	1.0	2012	http://sourceforge.net/projects/glmnb/	N/A	[62]	
Hpeak (Hidden Markov model (HMM)-based Peak-finding algorithm)	HMM-based algorithm	Hidden Markov Model (HMM)	2010	Perl and C++	Linux, Windows, and Mac OS	N/A	V2.1	2009	http://www.sph.umich.edu/csg/qin/HPeak/	N/A	[63]	
MACS (Model-based analysis of ChIP-Seq)	MACS algorithm (use shift and sliding window algorithm)	Model-based Analysis of ChIP-Seq	2008	Python	Linux	stand-alone, no GUI, open source	1.4.2	2012	http://liulab.dfci.harvard.edu/MACS/	Yes	[64]	
NEXT-peak (the normal-exponential two-peak)	NEXT-peak algorithm	Normal-exponential two-peak (NEXT-peak) model	2013	C++	Linux	N/A	1.1	2013	http://ww2.odu.edu/~nxkim/nextpeak/	Yes	[65]	
PeakRanger	Same algorithm as PeakSeq for identifying broad regions. Summit-valley-alternator algorithm	Build the read coverage profile	2011	C++	Linux, Mac OS, and Windows	Support parallel cloud computing	1.16	2012	http://ranger.sourceforge.net/	Yes	[66]	
PeakSeq	PeakSeq - two-pass strategy	Two-pass strategy	2009	C and Perl	N/A	N/A	1.1	2011	http://info.gersteinlab.org/PeakSeq	N/A	[67]	
QuEST (Quantitative Enrichment of Sequence Tags)	Construct profiles and use shifting method	Statistical framework-Kernel Density Estimation approach	2008	C++	Linux, Mac OS	Open source, non-profit use	2.4	2009	http://mendel.stanford.edu/SidowLab/downloads/quest/	No	[68]	
SeqSite	Two-step strategy: detect tag-enriched regions and then pinpoint binding sites in the detected regions	Poisson model	2011	C/C++	Windows, Mac OS X, and Linux	Academic use only	1.1.2	2010	http://bioinfo.au.tsinghua.edu.cn/software/seqsite/	Yes	[69]	
SICER	Scoring scheme	Spatial clustering approach	2009	Python	Linux, Unix	N/A	v1.1	2011	http://home.gwu.edu/~wpeng/Software.htm	Yes	[70]	
SIPeS (Site Identification from Paired-end Sequencing)	SIPeS algorithm	Used dynamic fragment pileup value for peak calling	2010	C	Linux	Non-profit use	2.0	2010	http://gmdd.shgmo.org/Computational-Biology/ChIP-Seq/download/SIPeS	N/A	[71]	
SISSRs (Site Identification from Short Sequence Reads)	Site Identification from Short Sequence Reads (SISSRs) algorithm	Sliding window	2008	Perl	Linux, UNIX	N/A	v1.4	2008	http://sissrs.rajajothi.com/	N/A	[72]	
Sole-Search	Sole-Search program	Implemented several different analysis steps for peak calling	2010	Java	N/A	Web-based software	N/A	N/A	N/A	No	[73]	
T-PIC (Tree shape Peak Identification for ChIP-Seq)	Tree shape Peak Identification for ChIP-Seq (T-PIC) algorithm	Tree-based statistics	2011	R and Perl	N/A	N/A	One version	2011	http://www.math.miami.edu/~vhower/tpic.html	N/A	[74]	
USeq	Collection of algorithms and software for peak calling	Implemented several different methods for peak calling	2008	Java	Linux, Mac OS X, and Windows	GUI	8.6.6	2013	http://useq.sourceforge.net/	Yes	[75]	
W-ChIPeaks	PELT algorithm and BELT algorithm	Statistical methods control false discovery rate	2011	PHP, Perl, Java and C++	N/A	Web tool	1.0.1	2012	http://motif.bmi.ohio-state.edu/W-ChIPeaks/	Yes for BELT only	[76]	
ZINBA (Zero-Inflated Negative Binomial Algorithm)	Zero-Inflated Negative Binomial Algorithm (ZINBA)	Statistical framework	2011	C and R	Mac OS X and Linux/Unix	Support multi-core clusters	2.02.03	2012	http://code.google.com/p/zinba/	Yes	[77]	

The peak finding process contains three essential steps: pre-processing, mapping, and peak finding [78]. The pre-processing step removes erroneous and low quality reads. The mapping step maps the reads back to the reference genome. It is critical as multiple reads can be mapped to multiple locations in the genome. Thus, the mapping can be handled by increasing the specificity using unique reads only or increasing the sensitivity by allowing multiple alignments of reads. Finally, the peak finding step identifies significant peak signals among background signals [78].

Several algorithms have been developed for identifying true peaks. There are three types of peaks in ChIP-Seq data: punctate regions contain a few hundred base pairs or less, localized but broader regions contain up to a few kilobases, and broad regions contain up to several hundred kilobases. Different peak categories associate with different types of binding events. For example, punctate region is a signature of a sequence specific transcription factor such as NRSF or CTCF. A combination of punctate and broader regions associates with proteins such as RNA polymerase II. Broad regions can associate with histone marks and other chromatin domain signatures [79].

Different peak finding tools implement different algorithms for targeting these types of peaks. Thus, the users should select a peak finding tool that is relevant to the type of research being conducted for maximizing the chance obtaining the best possible peak sequences for finding the motifs. There are software tools such as peakROTS [80] and the tool presented by Schweikert et al. [81] that are capable for assisting the users for optimizing the peak calling as well as choosing relevant software package for their analysis. The users may need to consider these tools. Here we provide an overview for each tool and hope the users may find it useful.

peakROTS implemented a generic data-adaptive procedure that allows to optimally adjust the parameters of a given software package to the properties of each ChIP-Seq dataset independently. It allows avoiding poor parameter settings for a given dataset. It can provide direction for selecting peak calling parameters. It notifies the users whether or not the quality of the data and/or the software parameters of a selected software package are sufficient for reliable binding site detections. It also recommends the users to choose the package that is optimal for a given dataset [80].

Schweikert et al. presented a tool, which implemented a combination and fusion analysis method. This tool provides a general assessment of available technologies and systems for assisting researchers to select a suitable system for their ChIP-Seq analysis. It also offers an alternative approach for increasing true positive rates and decreasing false positive rates. The tool can take different peak sequence outputs of the same dataset generated by different peak calling tools. It analyzes these peak sequence outputs and combines them in such a way that it can produce a better output from all peak sequence outputs it analyzes. Then, the improved peak sequence output can be used for further analysis [81].

### Results and discussion

#### ***Datasets***

We used five datasets from ChIP-Seq experiments in Shen et al. [82] in Table 3 for our motif discovery. These datasets came from mouse liver tissues, which have been sequenced on Illumina Genome Analyzer II and aligned to the mouse reference genome mm9. The output alignments are in bam format [82].

**Table 3:** Dataset’s properties

**Dataset**	**Mark**	**GEO accession**	**Number of sequences**	**Shortest sequence (residues)**	**Longest sequence (residues)**	**Total length (residues)**	**Size (FASTA format)**	**Size (BED format)**	**Reference**
DM230	PolII (RNA polymerase II)	GSM722763	105	157	1728	47242	49 KB	5 KB	[82]
DM05	p300 (co-activator protein)	GSM722762	142	130	1214	50318	53 KB	7 KB	[82]
DM254	CTCF (insulator binding protein)	GSM722759	4009	94	2374	1518265	1604 KB	181 KB	[82]
DM01	H3K4me1 (histone H3 lysine 4 monomethylation)	GSM722760	2001	175	8520	1856431	1871 KB	88 KB	[82]
DM721	H3K27ac (H3 lysine 27 acetylation)	GSM851275	4005	255	16542	5429909	5423 KB	180 KB	[82]

We ran MACS [64] on each dataset for obtaining the output peak file in bed format using P-value cutoff 0.00001 for peak detection. However, these peak sequence datasets are large and different motif finding Web tools accept the datasets with different limited sizes. Thus, we reduced the size of these datasets appropriately so that they can be accepted by the motif finding Web tools. In addition, each motif finding Web tool accepts different formats for peak sequence dataset. Therefore, we prepared the format for each peak sequence dataset appropriately for each motif finding Web tool. We used a utility BED to fasta conversion from CompleteMotifs [8] for converting the peak sequence outputs from MACS to fasta format for the Web tools that only accept fasta format. The details for each dataset are in Table 3.

#### ***Results***

We used two small datasets DM230 and DM05 in fasta format for running MEME [4], GLAM2 [5], W-ChIPMotifs [7], and CompleteMOTIFs [8] as these tools are unable to process large datasets. The parameters used for running MEME for both datasets are in Additional file 1: Table S1. We used all default parameters provided by GLAM2 for running both datasets. These parameters can be found in Additional file 1: Table S2. For running W-ChIPMotifs, we selected mouse species and left the transcription factor blank for running both datasets. For running CompleteMOTIFs, we used the parameters in Additional file 1: Table S3 for both datasets. As of this writing, the motif finding jobs for both datasets have not completed by Complete MOTIFs although these jobs have been submitted for over two months.

We used all five datasets in fasta format for running CisFinder [6], DREME [9], MEME-ChIP [10], and peak-motifs [11]. The parameters used for running all five datasets for these Web tools are in Additional file 1: Tables S4, S5, S6 and S7. CisFinder produced a large number of motifs for each dataset as it can detect local over-represented motifs, alternative binding motifs, binding motifs of potential co-factors, and motifs with a low level of enrichment.

We also used all five datasets in bed format for running PScanChIP [3]. The parameters used for running these datasets are in Additional file 1: Table S8. PScanChIP outputted all global and local over-represented motifs with their global and local P-values for each motif. We used P-value ≤ 0.05 as a threshold for filtering both global and local over-represented motifs in the results. The number of global over-represented motifs and local over-represented motifs after applying the filter for each dataset are in Additional file 1: Table S9. A summary of all results reported by each Web tool are also in Additional file 1: Table S9.

#### ***Discussion***

It is difficult to compare motif results from different motif finding tools even for the same peak sequence dataset because of the following reasons. Different motif finding Web tools implement different algorithms, which determine the results of the motif finding. In addition, each motif finding Web tool has its own parameters set up for finding motifs. The default parameters and the parameters selected by the users have an influence on the motif results. Thus, Tompa et al. suggested using a combination of different motif finding tools for maximizing the chance obtaining significant motifs [83]. Moreover, motifs reported by multiple tools are more reliable. On the other hand, multiple motif finding tools that implement different algorithms report identical motifs for the same dataset prove the consistency and reliability of these tools. However, in reality it is hard for these motif finding tools to agee on the same set of motifs that are exactly matched. Thus, we looked for similarities between these motifs reported by different motif finding tools. We used STAMP [43] for this purpose by comparing the similarities between two set of motif results from two different motif finding Web tools. We implemented this pair-wise comparison for all motif finding Web tools for each dataset. Since STAMP has its own required input formats and the formats of the motif results from different motif finding Web tools vary, we prepared the motif results in the formats required by STAMP for running this tool. Besides, different motif finding Web tools provide different settings for getting the maximum number of motifs to return. Thus, we obtained a variety number of motifs in the results from these tools for each dataset. Among them CisFinder reported the largest number of motifs. However, STAMP is not able to process large motif datasets. Hence, we reduced CisFinder’s motif datasets to ≤ 100 motifs in each dataset for STAMP to process.

We validated all motifs used for similarity comparisons with two reference databases: JASPAR [30] and UniProbe [33] for mouse species using TOMTOM [36] program with P-value cutoff ≤ 0.01. All discovered motifs in each dataset by MEME, GLAM2, W-ChIPMotifs, MEME-ChIP, and PScanChIP were found in either JASPAR or UniProbe. All discovered motifs by CisFinder for four datasets DM230, DM05, DM254, and DM721were found in either JASPAR or UniProbe except for one motif in the dataset DM01 was not found both databases. In addition, all discovered motifs by DREME for three datasets DM230, DM254, and DM721 were found in either JASPAR or UniProbe except for 2 motifs in the dataset DM01 were not found in both databases. Besides, RSAT peak-motifs showed two motifs that were not found in both references with one from the dataset DM254 and the other from the dataset DM01. All other discovered motifs by RSAT peak-motifs in other datasets were found in either JASPAR or UniProbe. In general, most of discovered motifs reported by each tool in each dataset used for similarity comparisons were found in the references for mouse species. All validation results can be found in column 4 of the Additional file 1: Table S11.

We performed the similarity comparisons as follows. For each Web tool, we compared its motif result with the motif result in every other Web tool for the same dataset using the matrix type in Additional file 1: Table S10. We performed this pair-wise comparison for all datasets for each Web tool. The pair-wise comparisons of motif results between these tools for the same dataset reveal the number of best matches by similarities between them. However, the resemble matches may not be one to one correspondence. The comparison results are in Additional file 1: Table S11. Most of discovered motifs by MEME were also found by CisFinder and W-ChIPMotifs. Besides, most of discovered motifs by GLAM2 were also found by all other tools except for MEME-ChIP.

For two small datasets DM230 and DM05, nearly all discovered motifs by CisFinder were also found by all other tools except for MEME-ChIP. For other three datasets, most of discovered motifs by CisFinder were also found by DREME and MEME-ChIP. However, peak-motifs and PScanChIP do not show a large number of similar motifs with CisFinder.

The output of W-ChIPMotifs includes the frequencies of nucleotides but they are not in the form of matrices. Thus, we converted these frequencies into raw PSSMs [84], which were used to compare with the motif results from other Web tools. Raw PSSM is defined in [84] as follows. It is an *l* × 4 matrix containing 4 columns for four nucleotides (A, C, G, T) and *l* rows for the size of the motif. Each entry in the matrix is the frequency value of a nucleotide in the multiple sequence alignments. The matrix is leaded by a character “>” followed by some characters, which can be the name of the matrix. The results show most of discovered motifs by W-ChIPMotifs were also found by MEME and CisFinder. However, other tools do not show a significant number of similar motifs with W-ChIPMotifs.

DREME returned only one motif for the dataset DM230. This motif was found by all other tools except for MEME-ChIP. DREME did not return any motif for the dataset DM05 although other tools reported a number of motifs for this dataset. For other three datasets, most of discovered motifs by DREME were also found by all other tools. However, PScanChIP does not show a large number of similar motifs with DREME.

MEME-ChIP integrated MEME and DREME into a pipeline, which maximizes the chance for obtaining the motifs that a single tool may miss because these tools are complement to each other. We used the parameter settings for this tool as used for running individual tool. However, MEME-ChIP did not report any motif for the dataset DM230 although MEME returned 20 motifs and DREME returned one motif. For the dataset DM05, MEME-ChIP returned 4 motifs, which were found by MEME but other tools do not show much similarities for this dataset. For other three datasets, most of discovered motifs by MEME-ChIP were also found by all other tools. However, PScanChIP does not show consistent motif similarities with MEME-ChIP.

For peak-motifs, most of discovered motifs by this tool for all datasets were also found by CisFinder. However, other tools do not show a lot similar motifs with peak-motifs.

PScanChIP does not allow exporting the motif results in matrix format for further analysis. To acquire the motif results in PSSMs format, we manually followed each motif’s link to the JASPAR database site for obtaining the corresponding matrix in JASPAR format for each motif. The comparison results show most of discovered motifs by PScanChIP for all datasets were also found by CisFinder. However, other tools do not show much similar motifs with PScanChIP.

In general, CisFinder shows consistent results comparing to the results from other tools as it produced a large number of motifs for each dataset. The capability to detect large number of motifs makes CisFinder consistent as some Web tools missed reporting the motifs that were found by others. We suggest the users to use multiple Web tools that implement different algorithms for their motif finding for obtaining significant motifs, overlapping resemble motifs, and non-overlapping motifs.

To date peak-motifs is the only Web tool that can take the input from other Web server via URL. This feature eliminates the uploading delay and speeds up the motif finding.

## Conclusions

In this work, we surveyed nine motif finding Web tools that are capable for finding binding site motifs. For each Web tool, we observed its features, approach, strengths and weaknesses. We pointed out the results of motif finding depend on several factors and discussed the closest influence factor, which is the peak calling tool. We presented that different peak calling tools implement different algorithms for targeting different types of peaks. Thus, it is critical for the users to pick a suitable peak calling tool for the type of research being conducted so that it can maximize the chance for obtaining the best possible peak sequences for finding the motifs. We also presented the tools that are able to assist the users for optimizing peak calling result as well as for choosing relevant software package for their analysis. We also performed comparisons for nine motif finding Web tools using five different datasets from ChIP-Seq experiments. We showed that comparing motif results from different motif finding Web tools is difficult because each tool has its own parameter settings as well as implementing different algorithms for finding motifs. In addition, the default parameter settings and user’s selected parameters have an influence on the motif results. Thus, we compared the motif results from different motif finding Web tools based on their similarities using STAMP [43] tool. We performed pair-wise comparison between two set of motifs from two different Web tools for all datasets. The comparison results showed CisFinder reported consistent results comparing to other Web tools as it was able to detect a large number of motifs that were not reported by other Web tools. Since each motif finding Web tool has its own advantages for detecting motifs that other Web tools may not discover, we suggested the users to use multiple Web tools that implement different algorithms for obtaining significant motifs, overlapping resemble motifs, and non-overlapping motifs.

We observed that newer motif finding Web tools have the capability to find global over-represented motifs, local over-represented motifs, and alternative motifs. These newer tools can process large datasets with long sequences. We also observed that recent motif finding development tends to exploit the Web for providing ease of use to the users.

### Future work

From the observations above, we see that the future of the motif finding development for ChIP-Seq should be Web tool design with user-friendly interface. It should be developed as a pipeline system, which integrates a number of specialized motif finding tools for ChIP-Seq. Such system would allow the users to run a combination of specialized tools for maximizing the chance obtaining significant motifs, overlapping resemble motifs, and non-overlapping motifs. The future tool should be able to detect global over-represented motifs, local over-represented motifs, and alternative motifs. It should be able to process large datasets with long sequences generated from the NGS technology. The future tool should be able to take the input from other Web server via URL for circumventing the uploading delay and speeding up the motif detection. It is also a plus if the future tool can probe the user for the type of research being performed and provide advisory features prior to running the tool. Finally, the future tool should provide a number of convenient result formats for further analysis.

## Reviewers’ comments

### *First round*

#### *Reviewer’s report 1*

Prof. Sandor Pongor, International Centre for Genetic Engineering and biotechnology (ICGEB), Italy

The written English of the manuscript could be further improved. The subtitle “Graph” could be replaced by “Graph representations”, or “Graph Theory”.

*Author’s response:*

*We have revised the manuscript and further improved the language editing. We replaced “graph” with “graph representations” in the first paragraph of the Section *General approaches for motif finding and revised the subtitle “Graph” to “Graph representations” in this same section. Below are the changes in the manuscript.

*First paragraph of the Section *General approaches for motif finding:

*“…There are several different approaches for finding motifs such as profiles, consensuses, projection, graph representations, clustering, and tree-based*[2,13,14]*.”*

*In Section *General approaches for motif finding, subtitle Graph changed to Graph representations.

There are too many Tables. I suggest Tables 3 and 4 to be combined into one, and Tables 5 to 12 to go into Supplementary.

*Author’s response:*

We combined Tables 3 and 4 into one table named Table 3 and adjusted the text in the body of the manuscript referring to this table. We also moved Tables 5–12 to the Supplementary Tables file and renamed these tables to Supplementary Tables 1–11 respectively.

Quality of written English: Needs some language corrections before being published.

#### *Reviewer’s report 2*

Dr. Yuriy Gusev, Georgetown University Medical Center, USA

The manuscript presents a critical review of many of the existing motif finding tools and pipelines that are designed for the CHIP-seq data analysis. Many applications of NGS technologies including CHIP-seq applications has been steadily growing over past 5–8 years with ever growing amount of data generated across the globe. With the costs of next generation sequencing falling fast toward a $1000 mark per genome, many of the sequencing applications are becoming more accessible for researchers. There is a clearly identifiable need for effective, scalable and reproducible computational tools allowing for fast and cost effective processing and analysis of this vast amount of raw sequencing data.

The authors provided a detailed computational review and comparison of 9 published software packages for CHIP-seq data processing and analysis.

The results of their comparative analysis are clearly presented and provide, perhaps for the first time a survey of capabilities, advantages and limitations of the most current tools. One of the noticeable results of their study is that there is a dramatic difference in the output of these tools even thought the same data sets were analysed. An overlap reported in the paper is ranging from 0 to 100% that is a clear indication of a problem with the existing computational algorithms. The authors have proposed a computational criteria for selection of the best tool based on the largest number of binding motifs found for any particular data set. However, in reviewer opinion, it is clear that the results obtained with any particular tool might have high level of false positive results and purely computational approaches do not provide a clear path to avoid high level of false positive results. The biological validation might offer one of the solutions for this predicament however if the number of predicted binding sites is high the experimental validation might not be feasible.

Overall, this paper presents a timely and useful survey of CHIP-seq computational pipelines and while it might be of most interest for a relatively narrow community of bioinformaticians involved with NGS-seq data analysis, it is also could serve as a guide for the growing number of bio-medical researchers involved in translational and clinical applications of NGS technologies.

*Author’s response:*

We found that different Web tools reported different number of motifs even for the same dataset. Motifs reported by different Web tools that implement different algorithms are more reliable and we suggested the users to use multiple Web tools that implement different algorithms for obtaining overlapping motifs. The biological validation is one of the best ways to validate motifs but it may be impractical for high volume of motifs. We hope the number of overlapping motifs from multiple Web tools is feasible for this validation.

Quality of written English: Acceptable.

#### *Reviewer’s report 3*

Dr. Shyam Prabhakar (nominated by Prof. Limsoon Wong)

The manuscript “A survey of motif finding Web tools for detecting binding site motifs in ChIP-Seq data” by Tran and Huang reviews nine web tools for motif discovery. The authors describe the features of the tools and apply them to five mouse ChIP-seq datasets. They then quantify overlaps between the resulting motif lists. Finally, they suggest that multiple tools be applied to any individual data set, since each method has its own pluses and minuses.

Since there are many online motif discovery tools, it is certainly useful to have guidance on which tool one should use on any particular ChIP-seq dataset. The tool that’s best for histone ChIP-seq may not be the same as the one that’s best for TF ChIP-seq. Some tools may work well only when the dataset is relatively “clean,” and others may work under almost all conditions. Some may require tightly-defined binding regions, whereas others may tolerate broader regions extending beyond a thousand basepairs. Unfortunately, these issues are not addressed in any way. In fact, the manuscript provides no guidance at all on the quality of the predictions made by the various tools. At the end, one is still left wondering which tool(s) one should use. The only concrete recommendation is that it is better to use multiple tools, but in bioinformatics this is a platitude.

*Author’s response:*

We have presented the detailed features of each Web tool in the manuscript. For example, MEME suggests removing duplicate and low information sequences in the input dataset. MEME does not detect motifs for co-factors. However, other Web tools such as CisFinder, DREME, and PScanChIP are capable for detecting binding motifs for cofactor TFs. We have provided as much details as possible for the input dataset’s properties that each Web tool can accept. For instance, PScanChIP only processes100-150 bp around the center of the summit of the peak, MEME can take < 1000 bp for sequence’s length, and CisFinder can accept the sequence’s length ≤ 50 Mb produced by the peak caller. We hope these properties assist the users for deciding which tool is capable for processing short or broader regions. We have also provided the details of the output that each Web tool can provide, for instance, the size of the motif (short or longer) that each Web tool can detect and return.

All Web tools allow verifying discovered motifs with the reference. We have validated the discovered motifs reported by each tool in our similarity comparisons and found most of them exist in the reference databases (See our response to your suggestions section). However, some tools reported more motifs than others for the same dataset. Thus, we compared these tools for the motifs they reported on the same dataset. The comparison’s details, results, and discussions on the results reported by each Web tool are presented in the manuscript. Based on the comparison results we think it cannot be recommended which Web tool should be used for a particular ChIP-Seq dataset because there is no certainty to say precisely which tool is best for a particular ChIP-Seq dataset. Thus, we can only suggest the users to use multiple Web tools that implement different algorithms because the users can see exactly what they can get from each Web tool for their dataset and take appropriate action. This is also the approach that the pipeline motif detection tools implement which we discussed in the manuscript. We also suggested the users to obtain the overlapping motifs, which are more reliable because they are reported by different tools that implement different algorithms.

The section that lists the general features of each of the nine web tools seems too long. In the format sent to reviewers, it covers 12–13 pages. The features mentioned in this section are often not particularly noteworthy (“CisFinder’s output can be in HTML and text format”), and also frequently redundant because they are listed again in Table 2. This section could be shortened considerably.

*Author’s response:*

*We have provided as much details as possible to the readers so they can see the detailed features that each Web tool can provide. Table *2 contains a short summary of the features for each Web tool. We think this table can be used for a quick lookup.

The section on peak-calling tools is not well motivated. In general, everything upstream has an influence on motif discovery: the peak-caller, the binding landscape of the TF, the quality of the antibody used, noise in the ChIP-seq data set, read length, the read mapping protocol, the thoroughness with which repeats are masked, and so on. The list could be quite long, and it is not clear that peak calling is the most important factor, now that peak callers have become reasonably robust. I would not be surprised if the use of an inappropriate peak caller or an inappropriate pvalue threshold resulted in failure to discover relevant motifs. However, as far as I can tell, this manuscript does not provided any data or cite any papers that quantify the effect of peak calling on motif discovery.

*Author’s response:*

We mentioned some of the influence factors pointed out above in the revised manuscript and we focused only on the closest influence factor, which is the peak calling tool. We only presented the general idea that the result of peak calling tool used for finding motifs has an influence on the result of motif finding and suggested the users to consider software tools that are able to assist them for optimizing the peak calling results relevant to their data’s property. The suggested tools are discussed in the manuscript.

The five ChIP-seq data sets used to evaluate the motif discovery tools are problematic - it is not obvious in most cases what the correct motifs are (only one of the five is a DNA-binding TF). One could guess that the motifs of liver-specific TFs should be enriched in, say, H3K27ac peaks, but no attempt is made to check if this is the case, or to evaluate the algorithms in this way.

*Author’s response:*

As presented above, each Web tool allows verifying found motifs with one or more reference databases such as TRANSFAC or JASPAR using P-value or E-value threshold. We also validated the discovered motifs reported by each tool in our similarity comparisons and found most of them exist in the reference databases (See our response to your suggestions section). We rely on the correctness of TOMTOM and other methods that each Web tool used for verifying the motifs.

The Results section relies mainly on Table 15. This matrix-like table lists the proportion of motifs discovered by tool X that are also discovered by tool Y. The data in the table can be used to cluster the algorithms into groups by similarity. However, the similarity relationships could in many cases have been predicted in advance, because many of the web tools employ the same algorithms (MEME, Weeder) at the back end. Due to this sharing of back-end algorithms, multiple web tools could potentially identify the same incorrect motif.

*Author’s response:*

We have revised our suggestion for the users to use multiple Web tools that implement different algorithms because different Web tools, which implement different algorithms at the backend, report the same motifs for the same dataset are more reliable than a single Web tool. Although some Web tools implemented the same algorithms at the backend, they do not always report the same motifs. For example, MEME-ChIP integrated MEME and DREME into a pipeline. However, MEME-ChIP did not report any motif for the dataset DM230 although MEME reported 20 motifs and DREME reported one motif for the same dataset.

Suggestions:

It would have been more useful to start with ChIP-seq datasets for TFs that have known motifs (derived from protein-binding microarray data, for example), and then evaluate the web tools on their ability to recover the known motifs. Another possibility would be to evaluate the tools on the number of motifs they discover that match motifs contained in TRANSFAC or JASPAR. This latter approach is suitable if one is testing for co-motifs (motifs bound by TFs that co-bind with the ChIP-ed TF). However, it is vulnerable to artifacts – false GC-rich or AT-rich motifs frequently match TRANSFAC entries with the same nucleotide composition. Yet another suggestion would be to use cross-validation as a measure of motif quality/accuracy.

*Author’s response:*

*We validated all motifs used for our similarity comparisons with the references databases JASPAR and UniProbe for mouse species using TOMTOM program. Most of these motifs were found in either JASPAR or UniProbe with P-value ≤ 0.01. We rely on the correctness of TOMTOM and other methods that each Web tool uses for verifying found motifs with the reference databases. For example, MEME, GLAM2, DREME, and MEME-ChIP use TOMTOM. W-ChIPMotifs uses STAMP, and so on. We have added a validation paragraph to the subsection Discussion in the manuscript. Below is the additional paragraph (paragraph 2 of the subsection *Discussion).

*“We validated all motifs used for similarity comparisons with two reference databases: JASPAR*[30]*and UniProbe*[33]*for mouse species using TOMTOM*[36]*program with P-value cutoff ≤ 0.01. All discovered motifs in each dataset by MEME, GLAM2, W-ChIPMotifs, MEME-ChIP, and PScanChIP were found in either JASPAR or UniProbe. All discovered motifs by CisFinder for four datasets DM230, DM05, DM254, and DM721were found in either JASPAR or UniProbe except for one motif in the dataset DM01 was not found both databases. In addition, all discovered motifs by DREME for three datasets DM230, DM254, and DM721 were found in either JASPAR or UniProbe except for 2 motifs in the dataset DM01 were not found in both databases. Besides, RSAT peak-motifs showed two motifs that were not found in both references with one from the dataset DM254 and the other from the dataset DM01. All other discovered motifs by RSAT peak-motifs in other datasets were found in either JASPAR or UniProbe. In general, most of discovered motifs reported by each tool in each dataset used for similarity comparisons were found in the references for mouse species. All validation results can be found in column 4 of the Supplementary Table 11.”*

Minor issues not for publication:

1) Introduction: “Assume that a motif appears in each sequence, we have (n -l + 1)^t possible candidates for motifs.” To be more precise, perhaps this should be written as, “Assuming that exactly one motif appears in each sequence, …” Also, the authors should clarify that this statement applies only to the algorithms tested in this survey. It does not apply to thermodynamically based algorithms such as QPMEME, MatrixREDUCE and TherMoS, which use nonlinear optimization on a continuous space of affinity models.

*Author’s response:*

We have revised the definition of motif finding problem with more details in the manuscript. This is a simple definition, which may not apply to every algorithm discussed in this manuscript. The change made in the first paragraph of the section General approaches for motif finding in the manuscript is below.

*“…Motifs are short sequences of a similar pattern found in sequences of DNA or protein. Consider t input nucleotide sequences of length n and an array s* (*s*_1_, *s*_2_, *s*_3_, …, *s*_*t*_) *of starting positions with each position comes from each sequence. An alignment matrix is a matrix of t* × *l, which contains t sequences of starting positions from each sequence with length l where l is the size of an l-mer. A profile matrix is a matrix of 4* × *l containing 4 rows for four nucleotides (A, C, G, T) and l columns. Each entry in the profile matrix is the frequency of each nucleotide in the alignment matrix. The consensus score is the sum of highest frequencies from each column in the profile matrix. The motif finding problem can be stated simply as follows. Given t input nucleotide sequences of length n, we want to find a set of l-mers with one from each sequence such that they maximize the consensus score. Thus, we need to consider all* (*n*–*l*+1)^*t*^*possible starting positions or candidates for motifs. That is the number of candidates for motifs is exponential of the number of input sequences…”*

2) Motif finding Web tools section: “The EM algorithm has the following drawbacks … It assumes there is exactly one appearance of the shared motif appearing in each sequence of the dataset but this is not always the case.” The wording is a bit confusing here, because this is not really a drawback of EM *per se*. Rather, it is a drawback of one specific application of EM.

*Author’s response:*

We have clarified it in paragraph 3 of the subsection MEME in the manuscript as follows.

*“… The EM algorithm for motif finding presented by Lawrence* et al. *has the following drawbacks. It is not clear how to select the starting point and when to stop trying different starting points. It assumes there is exactly one appearance of the shared motif appearing in each sequence of the dataset but this is not always the case…”*

3) Same section: “MEME can only model a single motif at a time and it is unable to find alternative binding motifs or motifs for co-factors.” MEME should be able to find motifs for co-factors, because it masks previously discovered motifs when it looks for new motifs.

*Author’s response:*

We have revised it in the last paragraph of the subsection MEME in the manuscript as follows.

“Because MEME erases previous discovered motifs when it searches for new motifs, MEME can only model a single motif at a time and it does not detect alternative binding motifs, which are motifs for co-factors.”

4) Page 9: the acronym PSPM should be defined. More generally, many terms are used to describe binding affinity models (PSPM, PSSM, PWM, letter-probability matrix, Transfac matrix). As far as I could tell, some of these terms mean the same thing, at least as used in this manuscript.

*Author’s response:*

We have explained the acronym PSPM in paragraphs 3 and 4 of the subsection GLAM2 in the manuscript as follows.

*“…Other options in the HTML output include viewing alignment, viewing Position Specific Probability Matrix (PSPM), and finding replications that are similar to the best motif found*[5]*.*

*The PSPM is a 4* × *l matrix containing 4 rows for four nucleotides (A, C, G, T) and l columns where l is the size of the motif. Each entry in the matrix is the frequency of a nucleotide in the multiple alignments of the sequences. This frequency is represented by a probability value.”*

5) Results and Discussion, first sentence: “We used five datasets from ChIP-Seq experiments in Shen et al. [82] in Table 3 for our motif search.” “Motif search” should be replaced with “motif discovery.”

*Author’s response:*

We have revised this sentence as suggested. Below is the revised sentence in the subsection Datasets in the manuscript.

*“We used five datasets from ChIP-Seq experiments in Shen* et al. [82]*in Table 3 for our motif discovery.”*

6) Table 14: It’s not clear what is meant by “Raw PSSM.” The second column (matrix type) contains many different entries. How can the matrices be compared when the matrix type used for comparison is not the same? On the other hand, if the matrix type really is the same, could this column be left out?

*Author’s response:*

We have added a definition for raw PSSM in paragraph 5 of the subsection Discussion in the manuscript. We also directed the readers to a reference, which contains an URL of the site explaining this format. The change made in this paragraph is below.

*“The output of W-ChIPMotifs includes the frequencies of nucleotides but they are not in the form of matrices. Thus, we converted these frequencies into raw PSSMs* [84]*, which were used to compare with the motif results from other Web tools. Raw PSSM is defined in*[84]*as follows. It is an l* × *4 matrix containing 4 columns for four nucleotides (A, C, G, T) and l rows for the size of the motif. Each entry in the matrix is the frequency value of a nucleotide in the multiple sequence alignments. The matrix is leaded by a character “>” followed by some characters, which can be the name of the matrix…”*

Different matrix types can be compared with each other by STAMP tool as this tool accepts a wide variety of matrix formats. This flexibility allowed us to perform the comparisons presented in the manuscript. We included the matrix type column in this table for providing details of the comparisons to the readers. This table has been moved to the Supplementary Tables file and it was renamed to Supplementary Table 10.

7) Table 15: If I’m not mistaken, “N/A” should be replaced by “0” in Row 2, Column 8, which shows the MEME-DREME comparison.

*Author’s response:*

It was an error in the table. We have fixed this error by replacing “N/A” with “0 (0%)” in Row 2, Column 8. This error has been corrected for all rows and columns where the comparison between a dataset that has zero motif with another dataset that has zero or more motifs.

Quality of written English: Needs some language corrections before being published.

### *Second round*

#### *Reviewer’s report 1*

Prof. Sandor Pongor, International Centre for Genetic Engineering and biotechnology (ICGEB), Italy

Accepted.

Quality of written English: Acceptable.

#### *Reviewer’s report 2*

Dr. Yuriy Gusev, Georgetown University Medical Center, USA

I am satisfied with the authors response to my comments and recommend to accept the manuscript for publication.

Quality of written English: Acceptable.

#### *Reviewer’s report 3*

Dr. Shyam Prabhakar (nominated by Prof. Limsoon Wong).

The revised version fixes some of the issues raised in the first round of review. However, my main concern remains that the study provides no guidance on the quality of the predictions made by the various tools.

In the revised version, the authors have attempted to address this point by comparing the de novo predicted motifs against databases of known motifs: JASPAR and UniProbe. It is claimed that most of the predicted motifs exist in the reference databases, and therefore the predictions are valid. However, it is not clear if the database matching was done correctly. As far as I can tell, motifs were considered to have a database match if TOMTOM found a hit with raw P-value < 0.01. Because of the multiple testing problem (there are hundreds of motifs in the JASPAR + UniProbe database), this is actually a very loose P-value threshold. It corresponds to a false-discovery rate not far from 100%. In other words, even random, nonsense motifs would match the database at this P-value threshold. I submitted three random motifs as sample queries to TOMTOM: GSTWGR, AGACG and CMAWGT. These motifs were plucked out of thin air – as far as I know, they do not correspond to any real transcription factors. All three returned database matches with P < 0.01.

If the authors applied a false-discovery rate cutoff (TOMTOM q-value < 0.01, for example), it’s likely that only a fraction of predicted motifs would have database matches. This is because the number of motif predictions is too large – on average each tool predicted 34 motifs in one ChIP-seq dataset (average of Column 3 in Supplementary Table 11). Only the top few motifs in these lists are likely to be genuine.

*Author’s response:*

We have validated a few motifs using q-value cutoff < 0.01 on TOMTOM. We found this q-value cutoff resulted in losing motifs that we think they are significant because these motifs were reported by multiple tools. Below are some examples.

Example 1:

This motif below was found by MEME in the dataset DM05. It is motif number 17 in the list of total 46 motifs reported by MEME.

This motif was also found by GLAM2, CisFinder, W-ChIPMotifs, MEME-ChIP, peak-motifs, and PScanChIP. TOMTOM reported several matches for this motif with p-values < 0.01 but q-values are much larger than 0.01 (at least 0.188899 or greater at the time of this validation) for mouse species in JASPAR or UniProbe database. One example of these matches is Hoxc9_2367.2 (homeo box C9) for this motif in the UniProbe database for mouse with p-value = 0.00327818 and q-value = 0.6349. We also validated this motif again with STAMP and found that STAMP also reported the same match, which is Hoxc9_2367.2 (homeo box C9) for this motif in the UniProbe database with E-value = 3.5754e-02.

Example 2:

This motif below was found by MEME-ChIP in the dataset DM01. It is motif number 9 in the list of total 9 motifs reported by MEME-ChIP.

This motif was also found by CisFinder, peak-motifs, DREME, and PScanChIP. TOMTOM also reported several matches for this motif with p-values < 0.01 but q-values are much larger than 0.01 (at least 0.12092 or greater at the time of this validation) for mouse species in JASPAR or UniProbe database.

Example 3:

The motif below was found by peak-motifs in the dataset DM254. It is motif number 37 in the list of total 39 motifs reported by peak-motifs.

This motif was also reported by CisFinder, MEME-ChIP, DREME, and PScanChIP. Same as above, TOMTOM reported several matches for this motif with p-values < 0.01 but q-values > 0.01 (at least 0.354709 or greater at the time of this validation) for mouse species in JASPAR or UniProbe database.

The motifs in the examples above were found by multiple tools. TOMTOM found matches for these motifs in either JASPAR or UniProbe database for mouse with p-values < 0.01. We think these motifs are significant and should not be eliminated. However, the q-values reported by TOMTOM for these motifs exceed the stringent cutoff 0.01. Thus, if we applied this stringent cutoff q-value < 0.01 for the motifs in our similarity comparisons it would result in losing significant motifs.

I would suggest that more stringent cutoffs be applied at all stages of the analysis. It would probably help quite a bit to consider only the top motif predictions, and also to run TOMTOM with a q-value threshold rather than a P-value threshold. I am not completely clear on how STAMPY was applied in this study, but it would be important to apply a q-value cutoff there as well.

*Author’s response:*

Using more stringent cutoffs can eliminate false positives. However, these stringent cutoffs can also eliminate significant motifs. We do not have suggestion for the exact stringent cutoff that would balance both cases. Thus, we leave this value for the users to decide appropriately for their research.

We used STAMP for finding similar motifs that were reported by multiple tools using E-value cutoff ≤ 0.05 in our study. The results from STAMP provide similar motifs using E-value only. Therefore, we can only use E-value for the cutoff.

## Competing interests

The authors declare that they have no competing interests.

## Authors’ contributions

NTLT designed, performed the experiments, and drafted the manuscript. C-HH directed and helped to draft the manuscript. All authors read and approved the final manuscript.

## Supplementary Material

Additional file 1 **Table S1.** Parameters selected for running MEME motif finding Web tool. **Table S2.** Parameters used for running GLAM2 motif finding Web tool. **Table S3.** Parameters used for running CompleteMOTIFs motif finding Web tool. **Table S4.** Parameters used for running CisFinder motif finding Web tool. **Table S5.** Parameters used for running DREME motif finding Web tool. **Table S6.** Parameters used for running MEME-ChIP motif finding Web tool. **Table S7.** Parameters used for running RSAT peak-motifs motif finding Web tool. **Table S8.** Parameters used for running PScanChIP motif finding Web tool. **Table S9.** A summary of the motif results for each dataset and Web tool. **Table S10.** Matrix types for motifs comparisons. **Table S11.** Comparing motif results between each motif finding Web tool with other motif finding Web tools for the number of best matched motifs using E-value ≤ 0.05 for each dataset. Click here for file
