# Supplementary material for: A survey of motif finding Web tools for detecting binding site motifs in ChIP-Seq data
Source: Biol Direct. 2014 Feb 20;9:4. doi: 10.1186/1745-6150-9-4 (PMC4022013; doi:10.1186/1745-6150-9-4)
Supplement: Additional file 1 — Table S1. Parameters selected for running MEME motif finding Web tool. Table S2. Parameters used for running GLAM2 motif finding Web tool. Table S3. Parameters used for running CompleteMOTIFs motif finding Web tool. Table S4. Parameters used for running CisFinder motif finding Web tool. Table S5. Parameters used for running DREME motif finding Web tool. Table S6. Parameters used for running MEME-ChIP motif finding Web tool. Table S7. Parameters used for running RSAT peak-motifs motif finding Web tool. Table S8. Parameters used for running PScanChIP motif finding Web tool. Table S9. A summary of the motif results for each dataset and Web tool. Table S10. Matrix types for motifs comparisons. Table S11. Comparing motif results between each motif finding Web tool with other motif finding Web tools for the number of best matched motifs using E-value ≤ 0.05 for each dataset. [file 1745-6150-9-4-S1.doc]

## Supplementary Tables

## Table S1 - Parameters selected for running MEME motif finding Web tool.

| **Parameter** | **Value** |
| --- | --- |
| Distribution of Motif occurrences | Zero or one per sequence |
| Number of different motifs (maximum number of motifs to find) | 10000 |
| Minimum motif width | 6 |
| Maximum motif width | 14 |

## Table S2 - Parameters used for running GLAM2 motif finding Web tool.

| **Parameter** | **Value** |
| --- | --- |
| Minimum aligned sequences | 2 |
| Minimum aligned columns | 2 |
| Maximum aligned columns | 50 |
| Initial aligned columns | 20 |
| Number of alignment replicates | 10 |
| Maximum iterations without improvement | 2000 |
| Deletion pseudocount | 0.1 |
| No-deletion pseudocount | 2 |
| Insertion pseudocount | 0.02 |
| No-insertion pseudocount | 1 |
| Examine both strands | Yes |
| Shuffle sequence letters | No |
| Embed sequences | Yes |

## Table S3 - Parameters used for running CompleteMOTIFs motif finding Web tool.

| **Parameter** | **Value** |
| --- | --- |
| Select an organism | mm9 |
| Select motif databases | Jaspar-CORE, Transfac |
| Type of background sequence | User input sequences (default) |
| Type of nucleotide shuffling | di-nucleotide (Markov order 1) (default) |
| Number of times for nucleotide shuffling | 1000 (default) |
| P-value cutoff | 0.05 (default) |
| De novo motif discovery | MEME (minimum motif width = 3 and maximum motif width = 14), Weeder (minimum motif width = 6 and maximum motif width = 12 by default), ChIPMunk (minimum motif width = 3 and maximum motif width = 13 by default) |

## Table S4 - Parameters used for running CisFinder motif finding Web tool.

| **Parameter** | **Value** |
| --- | --- |
| Use repeats for search | No |
| FDR | 0.05 |
| Count motif once per sequence | No |
| Minimum enrichment ratio (test versus control) | 1.5 |
| Match threshold for clustering | 0.75 |
| Clustering method | Similarity |
| Search in strands | Both |
| Adjust for CG/AT ratio and CpG | No |
| Score motifs by | Z+ratio |
| Maximum enrichment in repeats (ratio) | 1000 |
| Maximum number of motifs to find | 1000000 |

## Table S5 - Parameters used for running DREME motif finding Web tool.

| **Parameter** | **Value** |
| --- | --- |
| Select comparison source | Shuffle sequences (default) |
| Treatment of reverse complement strands | Use both strands (default) |
| E-value (expected number of false positives) | ≤ 0.05 (default) |
| Count (maximum number of motifs to find) | ≤ 1000000 |

## Table S6 - Parameters used for running MEME-ChIP motif finding Web tool.

| **Parameter** | **Value** |
| --- | --- |
| *Universal Option* |  |
| Scan both DNA strands | Use both strands (default) |
| *MEME Options* |  |
| Expected motif size distribution | Zero or one occurrence per sequence (default) |
| Maximum number of motifs to find | 1000000 |
| Minimum motif width | 6 |
| Maximum motif width | 14 |
| Minimum sites per motif | 2 (default) |
| Maximum sites per motif | 600 (default) |
| *DREME Options* |  |
| E-value (expected number of false positives) | ≤ 0.05 (default) |
| Count (maximum number of motifs to find) | ≤ 1000000 |
| *CentriMo Options* |  |
| Minimum acceptable match score | ≤ 5 (default) |
| E-value threshold | ≤ 10 (default) |
| Include sequence IDs | Yes (default) |

## Table S7 - Parameters used for running RSAT peak-motifs motif finding Web tool.

| **Parameter** | **Value** |
| --- | --- |
| *Information on the methods used in peak-motifs* |  |
| Mask | None (default) |
| *Reduce peak sequences* |  |
| Cut peak sequences | +/- 500 bp on each side of peak centers (default) |
| *Motif discovery parameters* |  |
| Discover over-represented words | Yes |
| Discover words with a positional bias | Yes |
| Oligomer length | 6, 7 (default) |
| Markov order (m) of the background model for oligo-analysis (k-mers) | Automatic (adapted to sequence length) (default) |
| Number of motifs per algorithm | 10 (maximum by default) |
| Search on | Both strands |
| *Compare discovered motifs with databases* |  |
| Compare discovered motifs with known motifs from databases | JASPAR core Vertebrates |
| *Locate motifs and export predicted sites as custom UCSC tracks* |  |
| Search putative binding sites in the peak sequences | Yes |
| Markov order | m = 1 |

## Table S8 - Parameters used for running PScanChIP motif finding Web tool.

| **Parameter** | **Value** |
| --- | --- |
| Organism | Mus musculus |
| Assembly | mm9 |
| Background | Mixed |
| Descriptors | Jaspar |

**Table S9 - A summary of the motif results for each dataset and Web tool.**

| **Tool** | **Dataset** | **Uploaded Format** | **Running Time** | **P-value/ E-value Selection** | **Number of Motifs Found** |
| --- | --- | --- | --- | --- | --- |
| MEME | DM230 | Fasta | 45 min 23 sec | N/A | 20 |
| MEME | DM05 | Fasta | 120 min | N/A | 46 |
| GLAM2 | DM230 | Fasta | 1 min 34 sec | N/A | 10 |
| GLAM2 | DM05 | Fasta | 1 min 25 sec | N/A | 10 |
| CisFinder | DM230 | Fasta | 1 sec | FDR = 0.05 | 185 (100 elementary motifs + 85 clusters of motifs) |
| CisFinder | DM05 | Fasta | 1 sec | FDR = 0.05 | 224 (142 elementary motifs + 82 clusters of motifs) |
| CisFinder | DM254 | Fasta | 1 min 9 sec | FDR = 0.05 | 2528 (2000 elementary motifs + 528 clusters of motifs) |
| CisFinder | DM01 | Fasta | 53 sec | FDR = 0.05 | 2642 (2000 elementary motifs + 642 clusters of motifs) |
| CisFinder | DM721 | Fasta | 60 sec | FDR = 0.05 | 1153 (1000 elementary motifs + 153 clusters of motifs) |
| W-ChIPMotifs | DM230 | Fasta | 67 min | N/A | 11 |
| W-ChIPMotifs | DM05 | Fasta | 76 min | N/A | 11 |
| CompleteMOTIFs | DM230 | Fasta | > 2 months | P-value = 0.05 | N/A |
| CompleteMOTIFs | DM05 | Fasta | > 2 months | P-value = 0.05 | N/A |
| DREME | DM230 | Fasta | 11 sec | E-value ≤ 0.05 | 1 |
| DREME | DM05 | Fasta | 5 sec | E-value ≤ 0.05 | 0 |
| DREME | DM254 | Fasta | 82 min 30 sec | E-value ≤ 0.05 | 45 |
| DREME | DM01 | Fasta | 112 min 30 sec | E-value ≤ 0.05 | 51 |
| DREME | DM721 | Fasta | 123 min 35 sec | E-value ≤ 0.05 | 16 |
| MEME-ChIP | DM230 | Fasta | 2 min 48 sec | E-value ≤ 0.05 | 0 |
| MEME-ChIP | DM05 | Fasta | 2 min 20 sec | E-value ≤ 0.05 | 4 (1 by DREME and 3 by MEME) |
| MEME-ChIP | DM254 | Fasta | 27 min | E-value ≤ 0.05 | 24 (21 by DREME and 3 by MEME) |
| MEME-ChIP | DM01 | Fasta | 16 min 30 sec | E-value ≤ 0.05 | 9 (6 by DREME and 3 by MEME) |
| MEME-ChIP | DM721 | Fasta | 22 min 50 sec | E-value ≤ 0.05 | 11 (8 by DREME and 3 by MEME) |
| RSAT peak-motifs | DM230 | Fasta | 2 min 30 sec | N/A | 10 |
| RSAT peak-motifs | DM05 | Fasta | 4 min 33 sec | N/A | 17 |
| RSAT peak-motifs | DM254 | Fasta | 14 min | N/A | 39 |
| RSAT peak-motifs | DM01 | Fasta | 17 min 50 sec | N/A | 40 |
| RSAT peak-motifs | DM721 | Fasta | 33 min | N/A | 40 |
| PScanChIP | DM230 | Bed | 3 sec | N/A | 14 unique global over-represented motifs (14 global over-represented motifs with global P-value ≤ 0.05, 0 local over-represented motif) |
| PScanChIP | DM05 | Bed | 3 sec | N/A | 16 unique global over-represented or local over-represented motifs (14 global over-represented motifs with global P-value ≤ 0.05, 2 local over-represented motifs with local P-value ≤ 0.05) |
| PScanChIP | DM254 | Bed | 23 sec | N/A | 63 unique global over-represented or local over-represented motifs (44 global over-represented motifs with global P-value ≤ 0.05, 60 local over-represented motifs with local P-value ≤ 0.05) |
| PScanChIP | DM01 | Bed | 12 sec | N/A | 27 unique global over-represented motifs (27 global over-represented motifs with global P-value ≤ 0.05, 0 local over-represented motif) |
| PScanChIP | DM721 | Bed | 25 sec | N/A | 37 unique global over-represented or local over-represented motifs (36 global over-represented motifs with global P-value ≤ 0.05, 2 local over-represented motifs with local P-value ≤ 0.05) |

## Table S10 - Matrix types for motifs comparisons.

| **Tool** | **Matrix Type** | **Sources** |
| --- | --- | --- |
| MEME | Position-specific probability matrices | MEME’s output |
| GLAM2 | Letter-probability matrices | GLAM2’s output |
| CisFinder | Raw PSSMs | CisFinder’s output |
| W-ChIPMotifs | Raw PSSMs | Manually converted nucleotide’s frequencies in the output to raw PSSMs |
| DREME | Letter-probability matrices | DREME’s output |
| MEME-ChIP | Letter-probability matrices | MEME-ChIP’s output |
| RSAT peak-motifs | TRANSFAC matrices | RSAT peak-motifs’s output |
| PScanChIP | Raw PSSMs | Manually collected from JASPAR database Web site |

## Table S11 - Comparing motif results between each motif finding Web tool with other motif finding Web tools for the number of best matched motifs using E-value ≤ 0.05 for each dataset.

| **Tool** | **Dataset** | **# of motifs** | **# of motifs found in JARPAR/UniProbe for Mouse (P-value ≤ 0.01)** | **MEME** | **GLAM2** | **CisFinder** | **W-ChIPMotifs** | **DREME** | **MEME-ChIP** | **RSAT peak-motifs** | **PScanChIP** |
| --- | --- | --- | --- | --- | --- | --- | --- | --- | --- | --- | --- |
| **MEME** | DM230 | 20 | 20 |  | 2 (10 %) | 20 (100 %) | 20 (100 %) | 1 (5 %) | 0 (0 %) | 16 (80 %) | 2 (10 %) |
| **MEME** | DM05 | 46 | 46 |  | 21 (46 %) | 42 (90 %) | 36 (78 %) | 0 (0 %) | 32 (70 %) | 23 (50 %) | 24 (52%) |
| **GLAM2** | DM230 | 10 | 10 | 10 (100 %) |  | 10 (100 %) | 10 (100 %) | 10 (100 %) | 0 (0 %) | 10 (100 %) | 10 (100 %) |
| **GLAM2** | DM05 | 10 | 10 | 10 (100 %) |  | 10 (100 %) | 8 (80 %) | 0 (0 %) | 5 (50 %) | 7 (70 %) | 7 (70 %) |
| **CisFinder** | DM230 | 85 | 85 | 73 (86 %) | 80 (94 %) |  | 84 (99 %) | 64 (75 %) | 0 (0 %) | 79 (93 %) | 79 (93 %) |
| **CisFinder** | DM05 | 82 | 82 | 81 (99 %) | 72 (88 %) |  | 66 (80 %) | 0 (0 %) | 26 (32%) | 77 (94 %) | 63 (77%) |
| **CisFinder** | DM254 | 100 | 100 | N/A | N/A |  | N/A | 100 (100 %) | 97 (97 %) | 66 (66 %) | 81 (81 %) |
| **CisFinder** | DM01 | 100 | 98 | N/A | N/A |  | N/A | 100 (100 %) | 89 (89 %) | 73 (73 %) | 55 (55 %) |
| **CisFinder** | DM721 | 100 | 100 | N/A | N/A |  | N/A | 99 (99 %) | 93 (93 %) | 77 (77 %) | 70 (70 %) |
| **W-ChIPMotifs** | DM230 | 11 | 11 | 10 (91 %) | 7 (64 %) | 11 (100 %) |  | 7 (64 %) | 0 (0 %) | 9 (82 %) | 7 (64 %) |
| **W-ChIPMotifs** | DM05 | 11 | 11 | 11 (100 %) | 7 (64 %) | 10 (91 %) |  | 0 (0 %) | 5 (45 %) | 4 (36 %) | 4 (36 %) |
| **DREME** | DM230 | 1 | 1 | 1 (100 %) | 1 (100 %) | 1 (100 %) | 1 (100 %) |  | 0 (0 %) | 1 (100 %) | 1 (100 %) |
| **DREME** | DM05 | 0 | 0 | 0 (0 %) | 0 (0 %) | 0 (0 %) | 0 (0 %) |  | 0 (0 %) | 0 (0 %) | 0 (0 %) |
| **DREME** | DM254 | 45 | 45 | N/A | N/A | 45 (100 %) | N/A |  | 45 (100 %) | 39 (87 %) | 42 (93 %) |
| **DREME** | DM01 | 51 | 49 | N/A | N/A | 50 (98 %) | N/A |  | 46 (90 %) | 50 (98 %) | 37 (73 %) |
| **DREME** | DM721 | 16 | 16 | N/A | N/A | 16 (100 %) | N/A |  | 15 (94 %) | 16 (100 %) | 11 (69 %) |
| **MEME-ChIP** | DM230 | 0 | 0 | 0 (0 %) | 0 (0 %) | 0 (0 %) | 0 (0 %) | 0 (0 %) |  | 0 (0 %) | 0 (0 %) |
| **MEME-ChIP** | DM05 | 4 | 4 | 4 (100 %) | 3 (75 %) | 2 (50 %) | 2 (50 %) | 0 (0 %) |  | 1 (25 %) | 2 (50 %) |
| **MEME-ChIP** | DM254 | 24 | 24 | N/A | N/A | 24 (100 %) | N/A | 24 (100 %) |  | 22 (92 %) | 23 (96 %) |
| **MEME-ChIP** | DM01 | 9 | 9 | N/A | N/A | 9 (100 %) | N/A | 9 (100 %) |  | 9 (100 %) | 4 (44 %) |
| **MEME-ChIP** | DM721 | 11 | 11 | N/A | N/A | 11 (100 %) | N/A | 11 (100 %) |  | 10 (91 %) | 10 (91 %) |
| **RSAT peak-motifs** | DM230 | 10 | 10 | 6 (60 %) | 2 (20 %) | 7 (70 %) | 6 (60 %) | 2 (20 %) | 0 (0 %) |  | 4 (40 %) |
| **RSAT peak-motifs** | DM05 | 17 | 16 | 14 (82 %) | 8 (47 %) | 15 (88 %) | 9 (53 %) | 0 (0 %) | 3 (18 %) |  | 16 (94 %) |
| **RSAT peak-motifs** | DM254 | 39 | 38 | N/A | N/A | 33 (85 %) | N/A | 39 (100 %) | 35 (90 %) |  | 29 (74 %) |
| **RSAT peak-motifs** | DM01 | 40 | 39 | N/A | N/A | 32 (80 %) | N/A | 38 (95 %) | 19 (48 %) |  | 12 (30 %) |
| **RSAT peak-motifs** | DM721 | 40 | 40 | N/A | N/A | 32 (80 %) | N/A | 33 (83 %) | 31 (78 %) |  | 13 (33 %) |
| **PScanChIP** | DM230 | 14 | 14 | 4 (29 %) | 7 (50 %) | 14 (100 %) | 11 (79 %) | 9 (64 %) | 0 (0 %) | 10 (71 %) |  |
| **PScanChIP** | DM05 | 16 | 16 | 15 (94 %) | 10 (63 %) | 16 100 %) | 9 (56 %) | 0 (0 %) | 8 (50 %) | 13 (81 %) |  |
| **PScanChIP** | DM254 | 63 | 63 | N/A | N/A | 58 (92 %) | N/A | 60 (95 %) | 55 (87 %) | 36 (57 %) |  |
| **PScanChIP** | DM01 | 27 | 27 | N/A | N/A | 25 (93 %) | N/A | 26 (96 %) | 15 (56 %) | 20 (74 %) |  |
| **PScanChIP** | DM721 | 37 | 37 | N/A | N/A | 37 (100 %) | N/A | 28 (76 %) | 20 (54 %) | 27 (73 %) |  |
